# Lorenz Plot Analysis in Dogs with Sinus Rhythm and Tachyarrhythmias

**DOI:** 10.3390/ani11061645

**Published:** 2021-06-01

**Authors:** Giovanni Romito, Carlo Guglielmini, Helen Poser, Marco Baron Toaldo

**Affiliations:** 1 Department of Veterinary Medical Sciences, Alma Mater Studiorum—University of Bologna, 40064 Bologna, Italy; giovanni.romito2@unibo.it (G.R.); marco.barontoaldo@unibo.it (M.B.T.); 2 Department of Animal Medicine, Production and Health, University of Padova, 35020 Padua, Italy; helen.poser@unipd.it

**Keywords:** electrocardiography, Holter, Poincaré plot, heart rate variability, canine

## Abstract

**Simple Summary:**

The Lorenz plot (LP) is a geometrical method to assess the dynamics of heart rate variability. It consists of a two-dimensional Cartesian coordinate system derived from electrocardiographic monitoring, in which each recorded R-R interval is plotted as a function of the previous R-R interval, and the values of each pair of successive R-R interval define a dot in the plot. The resultant clusters of dots can be evaluated quantitatively and qualitatively, and categorized into distinct geometrical patterns. In humans, several studies have demonstrated that the analysis of LP patterns (LPPs) has the potential to speed-up and improve the accuracy of arrhythmia detection and differentiation, especially in patients with tachyarrhythmias. As data on LP analysis are limited in dogs, this study describes the graphic features of LP derived from Holter recordings obtained in dogs with sinus rhythm and tachyarrhythmias, and analyzes the usefulness of LPP recognition in this species. We sought to evaluate if distinct cardiac rhythms imprint distinct and reproducible LPPs in dogs, as previously described in humans, and if each LLP can be used as a sensitive and specific indicator of a particular cardiac rhythm in this species.

**Abstract:**

The Lorenz plot (LP), a graphical representation of heart rate variability, has been poorly studied in dogs to date. The present study aimed to describe the graphic features of LP in dogs with sinus rhythm (SR) and tachyarrhythmias, and to analyze the usefulness of its pattern recognition. One hundred and nineteen canine Holter recordings were retrospectively evaluated. Cardiac rhythms were classified as: SR; SR with frequent (>100) premature ectopies (atrial, SR-APCs; ventricular, SR-VPCs; atrial and ventricular, SR-APCs-VPCs); atrial fibrillation (AF); and AF with frequent VPCs (AF-VPCs). Lorenz plots were studied qualitatively and quantitatively, and classified by distinct LP patterns (LPPs). Repeatability and reproducibility of LPP classification and diagnostic value were determined. Recordings included: 48 SR, 9 SR-APCs, 35 SR-VPCs, 5 SR-APCs-VPCs, 4 AF, and 18 AF-VPCs. Ten LPPs were identified: comet (*n* = 12), torpedo (*n* = 3), Y-shaped (*n* = 6), diamond (*n* = 10), diamond with a central silent zone (*n* = 17), double side-lobe (DSL) (*n* = 47), triple side-lobe (*n* = 1), quadruple side-lobe (*n* = 2), fan (*n* = 18), and fan with DSL (*n* = 3). Repeatability and reproducibility of LPP classification were excellent. The DSL pattern was both highly sensitive (91.3%) and specific (94.5%) for SR with frequent premature ectopies, either APCs, or VPCs, or both. The remaining LPPs had lower diagnostic value (high specificity but low sensitivity). Distinct rhythms imprint distinct and reproducible LPPs in dogs. The majority of canine LPPs are specific but insensitive indicators of SR and tachyarrhythmias.

## 1. Introduction

A variety of Holter software algorithms have emerged in the last decades to speed-up rhythm analysis and simplify the interpretation of complex cardiac rhythms. Lorenz plots (LP), also known as Poincaré plots or scatterplots, provide visual representation of oscillations in the period between consecutive heartbeats, by plotting each R-R interval against the next one in a two-dimensional Cartesian coordinate system and depicting it as a dot. In this way, recorded beats are organized into clusters of dots with particular shapes, sizes, and positions according to the R-R interval fluctuations and the underlying cardiac rhythm [1,2]. In humans, analysis of such clusters in patients with sinus rhythm (SR) and tachyarrhythmias has allowed for the identification of 10 distinct LP patterns, each one having its own specific graphic characteristics, nomenclature, and excellent diagnostic accuracy for rhythm diagnosis [2]. Therefore, LPs could represent an attractive diagnostic tool in veterinary cardiology as well. Regrettably, only a few studies have described the graphic features of LP in dogs with and without arrhythmias [3,4,5,6,7], and the only study aimed at characterizing the LP patterns in arrhythmic dogs evaluated LPs generated from 1-h Holter recordings [6].

Therefore, the purposes of this study are threefold: (1) to evaluate the graphic characteristics of LPs derived from long-term Holter analysis obtained from dogs with SR and tachyarrhythmias; (2) to assess the reproducibility of LP pattern classification; and (3) to determine the diagnostic performance of LP patterns in identifying different cardiac rhythms. We hypothesized that distinct cardiac rhythms would imprint distinct and recognizable LP patterns in dogs.

## 2. Materials and Methods

### 2.1. Study Population

Holter recordings from all dogs that underwent a Holter analysis as part of the diagnostic evaluation at the veterinary teaching hospitals of the University of Bologna and Padova between June 2006 and January 2019 were retrospectively reviewed. Holter recordings belonged to dogs with cardiac and/or extra-cardiac disorders associated with rhythm irregularities detected during cardiac auscultation and/or surface electrocardiogram, or to clinically healthy dogs of predisposed canine breeds (e.g., Doberman Pinschers) screened for dilated cardiomyopathy. Inclusion was restricted to good quality Holter recordings with ≥20 h of valid data and a Holter diagnosis of SR or tachyarrhythmias, either supraventricular or ventricular. For the purpose of this study, Holter recordings obtained from dogs with atrial fibrillation (AF) were included only if this tachyarrhythmia was observed for the entire duration of the monitoring. Recordings showing advanced or complete atrioventricular blocks, and those consistent with sick sinus syndrome, were excluded. Information regarding signalment, underlying cardiac or extracardiac disorders, and ongoing cardiac treatment at the time of Holter recording were obtained from medical records.

### 2.2. Holter Monitoring and Rhythm Analysis

Holter recordings were obtained at the two centers using the same standardized technique [8] and analyzed using a commercially available software program designed for humans (Cube Holter software, Cardioline S.p.A.). The monitoring was conducted in the dog’s home environment during their daily routines, and the owners were instructed to note all activities in a diary. Holter data were acquired with a resolution of 10-bit and at a sampling frequency of 250 Hz and were then transferred to a computer for analysis. A standard protocol for semiautomatic arrhythmia analysis was performed as previously described [9,10,11]. Initially, a single board-certified cardiologist (GR) manually checked the entire recording to assess its quality, ensure that the software triggered correctly on every complex, and label possible unidentified complexes. Based on R-R intervals, the software automatically calculated minimum, mean, and maximal heart rates. Subsequently, events marked by the software were manually checked by the same operator to confirm correct classification. The software distinguished QRS complex of sinus and supraventricular origin (N) from wide QRS complexes (‘atypical’). The software defined more than 3 successive N at a heart rate <60 beats per minute as bradycardia, and N-N intervals >2 s as sinus pauses. The software also noted events with an NN interval at least 80% shorter than the previous N-N interval. These events were manually differentiated into atrial premature complexes ([APCs], i.e., abnormal P waves conducting normal-appearing QRS complexes [12,13]) and premature normal complexes (i.e., premature sinus complexes during sinus arrhythmia; the latter was defined as a regularly irregular SR, which is characterized by cyclic variations of the R-R intervals and is associated with a rate appropriate for temperament and level of activity of the dog [9,10,11]). Atypical complexes were manually checked to confirm if they were ectopic complexes of ventricular origin (i.e., wide and abnormal looking QRS not associated with sinus P waves [12,13]). The software defined atypical complexes with a coupling interval at least 5% shorter than the previous N-N interval as ventricular premature complexes (VPCs). For the purposes of this study, only the number of APCs and VPCs, and not their organization into atrial and ventricular allorhythmias, was considered. Presence of AF entirely relied on manual analysis and was based on standard criteria (i.e., replacement of isoelectric baseline and sinus P waves by sequential less-defined deflections varying in amplitude, morphology and cycle length, associated with normal-appearing QRS complexes, and irregular ventricular rhythm [12,13]). In light of arrhythmias analysis, the same operator made a final rhythm diagnosis for each recording.

For statistical analysis, given the wide range of electrocardiographic abnormalities identified on Holter recordings, cardiac rhythms were arbitrarily divided into the following classes: (1) SR: SR (with/without phases of SR arrhythmia) as dominant rhythm throughout the recording, including both pure SR and SR associated with infrequent (<100/Holter [14,15]) APCs or VPCs; (2) SR-APCs: SR associated with frequent (>100/Holter) APCs, both isolated and organized into atrial allorhythmias; (3) SR-VPCs: SR associated with frequent VPCs (>100/Holter), both isolated and organized into ventricular allorhythmias; (4) SR-APCs-VPCs: SR associated with frequent APCs and VPCs (in both cases, >100/Holter), both isolated and organized into atrial and/or ventricular allorhythmias; (5) AF: AF as dominant rhythm throughout the recording, including both pure AF and AF associated with infrequent VPCs (<100/Holter); and (6) AF-VPCs: AF associated with frequent VPCs (>100/Holter), both isolated and organized into ventricular allorhythmias. In Holter recordings from the last class, particular attention was paid to manually differentiate true VPCs from QRS-complex changes related to rate-dependent aberrancy due to the Ashman phenomenon. Such differentiation was primarily based on the following electrocardiographic criteria: (1) the coupling interval of the wide QRS complex with the previous beat (true VPCs typically have a fixed coupling interval, while the Ashman phenomenon systematically results from a long R-R/short R-R sequence); (2) the presence or absence of a pause after the wide QRS complex (true VPCs are typically followed by a post-extrasystolic pause, whereas such pause is not expected in the case of the Ashman phenomenon); (3) the morphology of the wide QRS complex (the morphology of true VPCs can vary over the same recording due to the occurrence of VPCs arising from different ectopic foci, whereas the QRS-configuration tends to be stable in the case of Ashman phenomenon); and (4) the tendency of the wide QRS complex beats to form groups (VPCs tend to organize in couplets, triplets, or bigeminy, whereas such type of organization is atypical for the Ashman phenomenon) [16].

### 2.3. Heart Rate Variability Analysis

The software allowed analysis of heart rate variability (HRV), including both time- and frequency-domain analysis. Complexes of ventricular origin were automatically excluded from HRV analysis. Consequently, NN intervals included in the HRV analysis comprised sinus complexes and APCs [9,10,11]. For the purposes of this study, only time-domain variables were considered, including: the standard deviation of all the N-N intervals; the standard deviation of the means of the N-N intervals for all 5 min segments; the mean of the standard deviations of all the N-N intervals for all 5 min segments; the percentage of interval differences of successive N-N intervals more than 50 ms; and the square root of the mean squared differences of successive N-N intervals [1,9,10,11]. The HRV of dogs from the SR class was categorized as ‘normal’ or ‘reduced’ based on data previously reported in the canine literature [17].

### 2.4. Lorenz Plot Analysis

**Figure 1:**
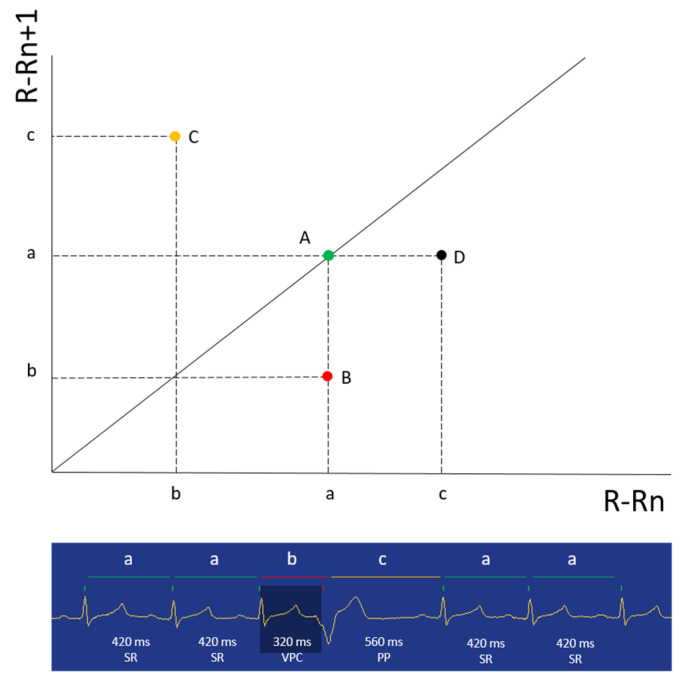
Example figure showing the basic principles of Lorenz plot generation. On the figure bottom, a selected portion of a Holter recording from this study shows a sinus rhythm with regular R-R intervals transiently interrupted by a ventricular premature complex with a post-ectopic pause (PP). Above the tracing, a two-dimensional Cartesian coordinates map of R-R intervals is illustrated. The X-axis is the R-Rn interval and the Y-axis is the R-Rn + 1 interval. In this example, the first two R-R intervals are regular (a), creating a dot on the diagonal line (identity line) (A). The third R-R interval is premature (b); therefore, the correspondent dot moves below the line to point B. The fourth R-R interval is longer due to the PP; thus, the correspondent dot moves above the line to point C. The fifth R-R interval returns to the initial value (a); consequently, the correspondent dot moves to point D. Since the last R-R interval is identical to the preceding one (a), the last dot returns to point A. Repetition of this process using the entire population of R-R intervals recorded during a Holter monitoring creates the final Lorenz plot.

Once the aforementioned steps were completed, the arrhythmia filter was disabled, and the complete population of recorded complexes was used to create LPs from the entire time of recording. On LPs, each R-R interval (R-Rn, dots depicted on the X-axis) was plotted against the following one (R-Rn + 1, dots depicted on the Y-axis), so that the coordinates of each dot were: R-R, R-R + 1 (Figure 1).

**Figure 2:**
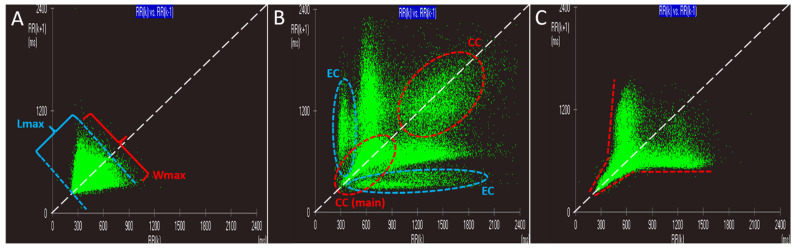
Graphic criteria used for classification of Lorenz plot patterns. (**A**): Example Lorenz plot generated from a Holter recording obtained from a dog with atrial fibrillation. Maximal length (Lmax) and maximal width (Wmax) of the cluster of dots are illustrated by blue and red lines, respectively. (**B**): Example Lorenz plot generated from a Holter recording obtained from a dog with sinus rhythm associated with a normal heart rate variability and frequent ventricular premature complexes. The central cluster (CC) and eccentric cluster (EC) are illustrated by red and blue dotted lines, respectively. Note that both CCs are located along the line of identity and that the main CC is placed at the lower left corner of the graph. Note also that both ECs arise from the lower left corner of the graph close to the body of the main CC and then extend outside the line of identity, one approximately parallel to the X-axis and the other approximately parallel to the Y-axis of the plot. (**C**): Example Lorenz plot from a Holter recording obtained from a dog with a diagnosis of sinus rhythm associated with a normal heart rate variability. Red dotted lines outline the margin of the CC and its diverging arms. Note that both arms are clearly attached to the CC, acting as its direct continuation and not as distinct ECs.

The resulting cloud of dots was characterized by: (1) a shape, fitted to the plot with the mean R-R interval as its center; (2) a length, the major axis passing through the mean R-R interval along the bisector (line of identity); and (3) a width, the minor axis passing through the mean R-R interval perpendicular to the major axis [1,2,5]. Then, as previously described in humans [2], each LP was studied qualitatively by assessing the specific shape of the contour of the main central cluster (CC) of dots, and also quantitatively by assessing three geometrical indexes: (1) the ratio of the maximal length to the maximal width; (2) the number of CCs and eccentric clusters (ECs) of dots; and (3) the number of branches arising from the main CC (Figure 2). Clusters of dots located along and outside the line of identity were defined as ‘central’ and ‘eccentric’, respectively [2]. When more than one CC was present, the term ‘main’ was used to define the cluster of dots located at the left lower corner of the graph. Table 1 reports the criteria of classification of LP patterns. The nomenclature already proposed in humans [2] and dogs [5,6,7] was used when the characteristics of patterns obtained from dogs included in the study were similar to those previously described; otherwise, a new nomenclature was proposed.

### 2.5. Statistical Analysis

Statistical analysis was performed with dedicated software (Microsoft Excel, version 2016, Microsoft Corporation; R, version 3.5.2, R Foundation for Statistical Computing, Vienna, Austria). The Shapiro–Wilk test was used to verify data distribution. Normally and non-normally distributed data were expressed as mean, standard deviation, and median (interquartile range), respectively. Repeatability and reproducibility of the classification of LP patterns were determined through intraobserver and interobserver agreement, respectively. The intraobserver agreement was determined from data generated by the same observer (GR) who evaluated blindly, four months apart, 20 randomly selected LPs. The interobserver agreement was determined from data generated by a second blinded observer (MBT) who analyzed the same 20 LPs used for intraobserver agreement. Intra and interobserver agreement were measured using the kappa statistics, as previously described [18]. The diagnostic value of LP analysis in identifying different rhythm classes was quantified in terms of sensitivity and specificity using standard formulas [19].

## 3. Results

### 3.1. Study Population and Holter Analysis

The study population included 119 Holter recordings from 102 client-owned dogs, with 11 animals undergoing more than one Holter monitoring. Sixteen (15.7%) and eighty-four (82.3%) dogs were clinically healthy and affected by cardiac or systemic diseases, respectively, whereas clinical diagnosis was not available in the remaining two (2%) dogs. Of the 119 Holter recordings, 62 (52.1%) were obtained from dogs without any cardiac treatment, 56 (47.1%) from dogs receiving at least one cardiac drug at the time of monitoring, whereas no data were available for one dog (0.8%) (Table 2).

The mean duration of Holter monitoring was 23.3 ± 1 h and all recordings had good quality ECGs that included the whole night. The SR was the most numerous class, followed by the SR-VPCs, and the AF-VPCs classes, whereas the SR-APCs, the SR-APCs-VPCs, and the AF classes were less frequent. Table 3 presents detailed information on rhythm classes and clinical diagnosis.

### 3.2. Classification, Repeatability and Reproducibility of Lorenz Plot Patterns

**Figure 3:**
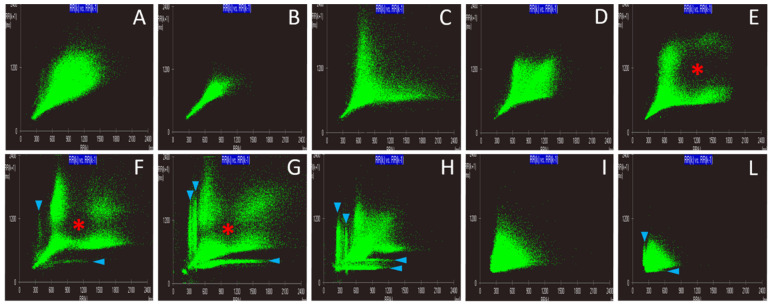
Lorenz plot patterns identified in the study population. (**A**): comet; (**B**): torpedo; (**C**): Y-shaped; (**D**): diamond; (**E**): diamond with a central silent zone; (**F**): double side-lobe (DSL); (**G**): triple side-lobe (TSL); (**H**): quadruple side-lobe (QSL); (**I**): fan; (**L**): fan with DSL. Red asterisks and blue arrowheads indicate the central silent zone and the eccentric clusters, respectively. Note that, in this figure, the DSL, TSL, and the QSL patterns are created by the association of the diamond with a central silent zone configuration with two eccentric clusters, the association of the diamond with a central silent zone configuration with three eccentric clusters, and the association of the diamond configuration with four eccentric clusters, respectively.

Lorenz plot analysis allowed for the identification of 10 distinct patterns (Figure 3 and Table 4). Features of six patterns were similar to those previously described in both humans and dogs, including comet, torpedo, Y-shaped, double side lobe (DSL), triple side lobe (TSL), and fan. The following nomenclature was proposed for the remaining four LP patterns: diamond, diamond with a central silent zone (CSZ) (i.e., a well-defined region of low density of dots within a PP pattern), quadruple side-lobe (QSL), and fan with DSL. The terms DSL, TSL, and QSL were used when one among the comet, torpedo, Y-shaped, diamond, or diamond with CSZ configuration was associated with two, three, and four ECs, respectively.

The intra- and interobserver agreement was high: K = 0.94 (standard error = 0.06; 95% confidence interval = 0.81–1.0) and K = 0.87 (standard error = 0.09; 95% confidence interval = 0.7–1.0), respectively.

### 3.3. Diagnostic Value of Lorenz Plot Patterns

Comet, torpedo, Y-shaped, diamond, and diamond with CSZ patterns were predominantly associated with SR (Table 4). Specifically, the torpedo pattern was associated with SR with reduced HRV, while comet, Y-shaped, diamond, and diamond with CSZ patterns were associated with SR and normal HRV (Table 5). The remaining patterns were mainly associated with tachyarrhythmias. Specifically, the DSL was predominantly associated with SR with frequent ectopic complexes, either APCs, VPCs, or both; the TSL and the QSL exclusively with SR with frequent VPCs; and the fan and the fan with DSL exclusively with AF, either with or without frequent VPCs (Table 4).

Comet, torpedo, Y-shaped, diamond, and diamond with CSZ patterns were poorly sensitive but highly specific in diagnosing the SR class. The DSL pattern appeared more sensitive than specific in diagnosing individually the SR-APCs, SR-VPCs, and SR-APCs-VPCs classes. However, when used to diagnose SR with frequent premature complexes irrespective of their origin (i.e., considering together the APCs, VPCs, and SR-APCs-VPCs classes), both sensitivity and specificity were high. The low number of TSL and QLS patterns limited individual statistical analysis, but when they were grouped together, low sensitivity and high specificity in diagnosing the SR-VPCs class were found. The fan pattern was more specific than sensitive in diagnosing both the AF and AF-VPCs classes. Similarly, the fan with DSL pattern was highly specific but poorly sensitive in diagnosing both the AF and AF-VPCs classes (Table 6).

## 4. Discussion

The main findings of the present study revealed that: (1) SR and tachyarrhythmias imprint peculiar signatures on canine LPs that can be categorized into 10 patterns; (2) the intra and interobserver agreement of LP patterns classification was excellent; and (3) the majority of LP patterns were highly specific but less sensitive indicators of canine SR and tachyarrhythmias.

Similar to humans [2,20,21,22,23], the comet and torpedo pattern were exclusively observed in dogs from the SR class, in particular in those with normal and reduced HRV, respectively. Graphically, comet and torpedo patterns had one CC characterized by a length bigger than the width. However, the former is narrow at the bottom and gradually gets wider towards the top along the line of identity showing a certain degree of asymmetry, and the latter maintains a relatively symmetric shape with a limited width. Human studies have demonstrated that an enhanced vagal cardiac activity leads to increased cluster width and asymmetry [2,20,21,22,23]. This can explain why dogs with SR and severely reduced HRV had a torpedo rather than a comet pattern.

As previously observed in healthy dogs [5,6,7], three other LP patterns predominantly occurred in the SR class with normal HRV, namely the Y-shaped, diamond, and diamond with CSZ. The Y-shaped pattern showed the most intense distribution of dots at the lower left corner of the graph, forming a stalk that extended from the X-axis at an angle of approximately 45° and splitting into two diverging arms approximately parallel to the X-axis and Y-axis of the plot. The stalk represents consecutive R-R intervals of a similar length (i.e., phases of regular SR), while the diverging arms are created by long-short and short-long R-R intervals (i.e., premature sinus complexes and sinus pauses during phases of sinus arrhythmia, respectively). Other LPs had a diamond configuration due to an additional cluster of dots filling the gap between the aforementioned arms. These dots represent consecutive R-R intervals with a longer length (e.g., phases of sinus bradycardia). Lastly, many LPs with a diamond configuration had a CSZ. This area represents a range of R-R intervals occurring infrequently when compared to others, and is likely due to the initiation of beats from different sinus node areas characterized by a fixed range of discharge rates or variation in the exit block from the sinus node under the physiologic control of the autonomic nervous system [7]. The reasons for these different LP patterns found in dogs from the SR class with normal HRV may depend on differences in breeds, age, sex, clinical status, and treatments [9,10,11,17,24,25,26].

Similar to human [2,27] and previous canine [6] studies, the DSL pattern was predominant in dogs with frequent ectopic complexes of any origin. Graphically, the human DSL pattern is composed by a central comet or torpedo configuration and two ECs [2]. Given the wide range of patterns associated with canine SR, we classified a DSL pattern when either a comet, torpedo, Y-shaped, diamond, and diamond with CSZ configuration was associated with two ECs. Eccentric clusters arose from the lower left corner of the graph, close to the body of the main CC, and then diverged distally, approximately parallel to the X-axis and Y-axis of the plot. The long-short R-R intervals produced by VPCs following sinus complexes, and the short-long R-R intervals produced by post-ectopic pauses following VPCs, created the right-sided and the left-sided ECs, respectively. Variable EC length, thickness, and density were observed in this study, as already reported in humans [2]. This finding likely reflects the variable number of premature ectopic complexes observed and the variable duration of their coupling intervals and post-ectopic pauses. In addition to the DSL pattern, an additional left-sided EC, creating a TSL pattern, was observed in a dog with SR + VPC. This pattern has been already described in humans and dogs with frequent VPCs and compensatory post-ectopic pauses [2,6,27]. Two dogs from this study, both from the SR-VPCs class, showed two left-sided and two right-sided ECs, creating a QSL pattern. This previously unreported pattern may derive from the coexistence of two ventricular foci, each one generating frequent VPCs with peculiar coupling intervals and stable duration of the relative post-ectopic pauses.

As in previous human [2,28] and canine [6] reports, the fan pattern was identified exclusively in dogs showing AF as the dominant cardiac rhythm. Graphically, this pattern shows widely scattered data dots organized into a single CC located along the line of identity. The shape of the most concentrated cluster resembles a triangle where the width is often bigger than the length, and the triangular apex is at the left lower corner of the graphs. Two dogs from the AF-VPCs class and one dog from the AF showed also two ECs, creating a fan with DSL pattern. Interestingly, the dog from the AF class had a number of VPCs (93 VPCs/Holter) close to the threshold beyond which the shift from the AF to the AF-VPCs class was set. Therefore, the VPCs of this dog were frequent enough to generate visible ECs. Intriguingly, 16/18 dogs from the AF-VPCs class had a fan rather a fan with DSL pattern. This can be explained by the relationship between the refractory period of the atrioventricular node during AF, and the prematurity of VPCs as well as the duration of their post-ectopic pauses. When coupling intervals of VPCs and duration of relative post-ectopic pauses significantly differ from R-R intervals recorded during AF, the resultant ECs should be clearly separated from the fan pattern envelope; conversely the fan pattern and ECs can overlap [29].

Regarding the reproducibility of LP patter classification, this study found an excellent intra and interobserver agreement. This finding is in line with the human literature, where good to excellent intra and interobserver agreement of LP pattern classification has been reported [2].

The majority of observed LP patterns were highly specific but less sensitive in identifying distinct rhythm classes; this was different from human LP patterns, which demonstrated both high sensitivity and specificity [2]. The reasons for this discrepancy are not immediately clear. Differences in study population size, rhythm classes analyzed, and software employed for LPs analysis can be a source of discrepancy [2]. Additionally, species-related peculiarities should be considered. For example, the discharge regularity of the human sinus node explains why human SR imprints only two patterns on LP (comet and torpedo) [2]. In contrast, the widely variable canine HRV led to five LP patterns in dogs from the SR class (comet, torpedo, Y-shaped, diamond, and diamond with CSZ), which inevitably reduce the sensitivity of each of them. Interestingly, the DSL pattern was both highly sensitive and specific in diagnosing SR with frequent premature ectopic complexes irrespective of their origin (i.e., considering together the APCs, VPCs, and SR-APCs-VPCs classes). This finding could bear relevance in small animal practice, especially in busy hospitals. Indeed, veterinary cardiologists often deal, over the same workday, not only with Holter analysis, but also with clinical and echocardiographic examinations, management of hospitalized patients, and interventional procedures. This can delay the Holter analysis completion, and, therefore, the start of antiarrhythmic treatment in some dogs with tachyarrhythmias. Therefore, one possible clinical implication of this result could be the strong justification to give priority to thorough Holter analysis when a DSL pattern is present, with the aim to start rapidly an antiarrhythmic treatment, if necessary.

This study has some limitations. First, the low frequency of some specific rhythm classes limited the statistical analysis of the relative LP patterns. Additionally, we did not include dogs with incessant tachyarrhythmias other than AF, which theoretically could generate a torpedo pattern. Moreover, only the number of ectopies, but not their organization into allorhythmias, was considered. This choice was purposefully taken, primarily to fill the information gap regarding the effect of ectopies on canine LP. We preferred to exclude from this initial study the analysis of the effect of the different types of organization of ectopies (e.g., bigeminy, trigeminy, or tachycardia) on LP in order to simplify this preliminary analysis, already rich in numerical and graphic data. However, it is among our future goals to perform such an analysis to expand further our comprehension on the effect of canine rhythm disturbances on LP. A second limitation relies on the variable duration of Holter recordings, ranging from 20–24 h. However, our recordings fulfilled the recommendations for HRV assessment in humans (i.e., at least 18 h of analyzable ECG data that includes the whole night) [1]. Third, we focused on examining the effects of frequent tachyarrhythmias on the LP shape, which implies that the effects of arrhythmic episodes with a low incidence could have been obscured by the more numerous sinus complexes. A fourth limitation could be related to our definition of ‘frequent’ ectopic complexes, which was based on previously reported canine cut-offs [14,15]. Moreover, it is important to note that an identical cut-off has been used in studies analyzing LPs in humans [2,27]. Lastly, given the lack of standardized and universally accepted thresholds for classifying canine HRV as ‘normal’ and ‘reduced’, classification of time-domain HRV variables of dogs from this study was arbitrary and based on comparison with a previous study [17]. Although that study only included dogs of a single breed (i.e., Doberman Pinschers), we anyway deemed appropriate to use it for comparison because it was the study analyzing the HRV parameters of our interest in the larger group of healthy dogs [17].

## 5. Conclusions

For the first time in dogs, we showed that SR and tachyarrhythmias imprint peculiar configurations on LPs that could be classified into a set of 10 patterns. Additionally, this study demonstrated an excellent reproducibility of LP pattern classification in dogs. The majority of LP patterns were highly specific but less sensitive in identifying SR and tachyarrhythmias in this species. Further studies are warranted to analyze LP patterns in dogs with different cardiac rhythms.

## Figures and Tables

**Table 1:** Quantitative and qualitative features of Lorenz plot patterns.

LP Pattern	Lmax/Wmax	CC (No.)	EC (No.)	Branches from CC (No.)	LP Morphology
Comet	>1	1	0	0	Comet/club
Torpedo	>1	1	0	0	Cigarette
Y-shaped	NA	1	0	2	Y
Diamond	1 or >1	1	0	0	Diamond/rhombus
Diamond with CSZ	1 or >1	2	0	0	Diamond/rhombus with a central rarefaction area
DSL	NA, <1, 1 or >1	1 or 2	2	0 or 2	One of the first five morphologies with two lateral fusiform ECs (one left-sided and one right-sided)
TSL	NA, <1, 1 or >1	1 or 2	3	0 or 2	One of the first five morphologies with three lateral small and fusiform ECs (two left-sided and one right-sided)
QSL	NA, <1, 1 or >1	1 or 2	4	0 or 2	One of the first five morphologies with four lateral small and fusiform ECs (two left-sided and two right-sided)
Fan	1 or <1	1	0	0	Triangular
Fan with DSL	1 or <1	1	2	0	Triangular with two lateral small and fusiform ECs (one left-sided and one right-sided)

CC: central cluster; CSZ: central silent zone; DSL: double side lobe; EC: eccentric cluster; Lmax/Wmax: ratio of the maximal length to the maximal width; LP: Loren plot; NA: not applicable; QSL: quadruple side lobe; TSL: triple side lobe.

**Table 2:** Demographic data from dogs used in this study.

Variable	
No. of dogs	102
Age (years)	10.0 (6.5–12)
Body weight (kg)	25.8 (13.8–35.0)
Sex (M/NM/F/NF)	49/8/22/23
Breed (No. of dogs)	Mixed breed (24)
	Doberman Pinscher (10)
	Boxer (9)
	German Shepherd, Labrador Retriever (5)
	English Bulldog, Golden Retriever (4)
	Cocker Spaniel, Corso (3)
	American Staffordshire Terrier, Cavalier King Charles Spaniel, English Pointer, Italian Hound, Pug (2)
	Basset Hound, Bolognese, Bouvier des Flandres, Briard, Czechoslovak Wolfdog, Dogo Argentino, English Setter, French Bulldog, Great Danes, Irish Setter, Irish Terrier, Jack Russell Terrier, Leonberger, Miniature Pinscher, Neapolitan Mastiff, Newfoundland, Nova Scotia Duck Tolling Retriever, Parson Russell Terrier, Poodle, Rottweiler, Shih Tzu, Vizsla, Weimaraner, West Highland White Terrier, Yorkshire Terrier (1)
Clinical status (H/D/NA)	16/84/2

D: systemic and/or cardiac disease; F: female; H: healthy; M: male; NA: not available; NF: neutered female; NM: neutered male.

**Table 3:** Electrocardiographic, clinical, and treatment data related to Holter recordings used in this study.

Holter Diagnosis (No.)	Clinical Diagnosis (No.)	Ongoing Cardiac Treatment (No.)
SR (48) (No. of VPCs: 2 (0–9))	cMMVD (16); AH (13); cardiac tumor, CHD (3); DCM, dMMVD, NA, suspected myocarditis, NM-TLoC, PH (2); Pheochromocytoma (1)	pimobendan (5); benazepril, furosemide (4); sotalol (3); amiodarone, atenolol, mexiletine (2); spironolactone (1)
SR-APCs (9) (No. of APCs: 2444 (1578–3502))	cMMVD (4); dMMVD (3); AH, non-specific LV cardiomyopathy (1)	benazepril, pimobendan (3); furosemide (2); amiodarone, amlodipine, hydrochlorothiazide, sildenafil, spironolactone, torasemide (1)
SR-VPCs (35) (No. of VPCs: 1589 (389–11,751))	cMMVD (8); DCM (6); suspected myocarditis (5); AC, CHD (4); cardiac tumor (3); AH, dMMVD (2); non-specific BV cardiomyopathy (1)	amiodarone (6); pimobendan (5); furosemide, sotalol (4); benazepril (3); atenolol, mexiletine (1)
SR-APCs-VPCs (5) (No. of APCs: 953 (332–3992)) (No. of VPCs: 1049 (175–2822))	dMMVD (2); cardiac tumor, DCM, suspected myocarditis (1)	benazepril, furosemide, pimobendan (4); amlodipine (2); atenolol, mexiletine (1)
AF (4) (No. of VPCs: (33 (8–65)	DCM (2); dMMVD, suspected myocarditis (1)	benazepril, furosemide, pimobendan (3); digoxin (2); diltiazem (1)
AF-VPCs (18) (No. of VPCs: 982 (366–2369))	dMMVD (9); cMMVD (4); DCM (3); HWD, CHD (1)	pimobendan (14); benazepril, furosemide (10); diltiazem (8); digoxin (7); torasemide (3); amlodipine, hydrochlorothiazide, spironolactone (1)

AC: arrhythmogenic cardiomyopathy; AF: atrial fibrillation; AH: apparently healthy; APC: atrial premature complex; BV: bi-ventricular; CHD: congenital heart disease; cMMVD: compensated myxomatous mitral valve disease (American College of Veterinary Internal Medicine stage B1 + B2); DCM: dilated cardiomyopathy; dMMVD: decompensated myxomatous mitral valve disease (American College of Veterinary Internal Medicine stage C + D); HWD: heartworm disease; LV: left ventricular; NA: not available; NM-TLoC: suspected neurally-mediated transient loss of consciousness; PH: pulmonary hypertension; SR: sinus rhythm; VPC: ventricular premature complex.

**Table 4:** Frequency of Lorenz plot patterns and related rhythm classes from Holter recordings used in this study.

Lorenz Plot Pattern (No.)	Rhythm Classes (No.)
Comet (12)	SR with normal HRV (12)
Torpedo (3)	SR with reduced HRV (3)
Y-shaped (6)	SR with normal HRV (6)
Diamond (10)	SR with normal HRV (8); SR-APCs (1); SR-VPCs (1)
Diamond with CSZ (17)	SR with normal HRV (15); SR-APCs (1); AF (1)
Double side lobe (47)	SR-VPCs (31); SR-APCs (7); SR-APCs-VPCs (5); SR with normal HRV (4)
Triple side lobe (1)	SR-VPCs (1)
Quadruple side lobe (2)	SR-VPCs (2)
Fan (18)	AF-VPCs (16); AF (2)
Fan with double side lobe (3)	AF-VPCs (2); AF (1)

AF: atrial fibrillation; APC: atrial premature complex; CSZ: central silent zone; HRV: heart rate variability; SR: sinus rhythm; VPC: ventricular premature complex.

**Table 5:** Time-domain heart rate variability parameters obtained from dogs of the sinus rhythm class, divided based on their Lorenz plot patterns.

HRV Variable	Lorenz Plot Pattern (No.)					Comparis. Intervals *
	Comet (12)	Torpedo (3)	Y-Shaped (6)	Diamond (10)	Diamond with CSZ (17)	
PNN50 (%)	43.7 ± 17.2	9.3 ± 4.2	34.0	51.0	48.0	56 ± 10
			(25.7–46.7)	(41.5–58.5)	(32.2–61.5)	(18–75)
RMSSD (ms)	148.8 ± 85.6	32.3 ± 6.1	130.5	174.0	204.0	325 ± 118
			(85.2–307.7)	(131.0–182.0)	(122.2-289.5)	(112-610)
SDANN (ms)	124.9 ± 64.5	72.0 (69.0–93.0)	145.5	120	131.0	195 ± 52
			(133.5–196.5)	(91.0–174.5)	(111.5–163.5)	(112–322)
SDNN (ms)	188.2 ± 95.3	84.0 ± 26.1	215.7 ± 111.0	204.0	208.0	331 ± 87
				(179.0–224.0)	(165.2–248.2)	(148–521)
SDNNIDX (ms)	155.0 ± 88.0	53.7 ± 15.3	143.0	164.6 ± 58.7	180.5	260 ± 76
			(111.7–238)		(122.2–215.5)	(88–415)

CSZ: central silent zone; HRV: heart rate variability; PNN50: percentage of interval differences of successive NN intervals more than 50 ms; RMSSD: square root of the mean squared differences of successive NN intervals; SDANN: standard deviation of the means of the NN intervals for all 5 min segments; SDNN: standard deviation of all the NN intervals; SDNNIDX: mean of the standard deviations of all the NN intervals for all 5 min segments. * Calvert, C.A.; Wall, M. Effect of severity of myocardial failure on heart rate variability in Doberman Pinschers with and without echocardiographic evidence of dilated cardiomyopathy [17].

**Table 6:** Diagnostic value of Lorenz plot patterns for the diagnosis of cardiac rhythms.

Lorenz Plot Pattern	Cardiac Rhythm	Se (%)	Sp (%)
		(95% CI)	(95% CI)
Comet	SR	25.0 (13.6–39.6)	100 (94.9–100)
Torpedo	SR	6.2 (1.3–17.2)	100 (94.9–100)
Y-shaped	SR	12.5 (4.7–25.3)	100 (94.9–100)
Diamond	SR	16.7 (7.5–30.2)	97.2 (90.2–99.7)
Diamond with CSZ	SR	31.2 (18.7–46.3)	97.2 (90.2–99.7)
DSL	SR-APCs	77.8 (39.9–97.2)	63.6 (53.9–72.6)
	SR-VPCs	88.6 (73.3–96.8)	81.0 (70.9–88.7)
	SR-APCs-VPCs	100 (47.8–100)	63.2 (53.6–72)
	SR with frequent ECs of any origin (APCs, VPCs or both)	91.3 (79.2–97.6)	94.5 (86.6–98.5)
TSL + QSL	SR-VPCs	8.8 (1.9–23.7)	100 (95.8–100)
Fan	AF	50.0 (6.8–93.2)	86.1 (78.4–91.8)
	AF-VPCs	88.9 (65.3–98.6)	98.0 (93–99.8)
Fan with DSL	AF	25.0 (0.6–80.6)	98.3 (93–99.8)
	AF-VPCs	11.1 (1.4–34.7)	99.0 (94.6–99.9)

AF: atrial fibrillation; APC: atrial premature complex; CI: confidence interval; CSZ: central silent zone; DSL: double side lode; EC: ectopic complex; QSL: quadruple side lobe; Se: sensitivity; Sp: specificity; SR: sinus rhythm; TSL: triple side lobe; VPC: ventricular premature complex.

## Data Availability

The data presented in this study are available on request from the corresponding author.
